# Improving the clinical recognition, prognosis, and treatment of melioidosis through epidemiology and clinical findings: The Sabah perspective

**DOI:** 10.1371/journal.pntd.0011696

**Published:** 2023-10-16

**Authors:** Ainulkhir Hussin, Mohd Yusof Nor Rahim, Frederick Dalusim, Muhammad Ashraf Shahidan, Sheila Nathan, Nazlina Ibrahim

**Affiliations:** 1 Department of Biological Sciences and Biotechnology, Faculty of Science and Technology, Universiti Kebangsaan Malaysia, Bangi, Selangor, Malaysia; 2 Department of Pathology, Queen Elizabeth Hospital, Ministry of Health Malaysia, Kota Kinabalu, Sabah, Malaysia; Yale University School of Medicine, UNITED STATES

## Abstract

**Introduction:**

Melioidosis is a deadly endemic disease in northern Australia and Southeast Asia, including Sabah, Malaysia, which is caused by the bacterium *Burkholderia pseudomallei*. It contributes to high fatality rates, mainly due to misdiagnosis leading to the wrong treatment being administered to the patients. Local epidemiology and data on clinical features could assist clinicians during diagnosis and treatment. However, these details are still scarce, particularly in Sabah.

**Methods:**

A retrospective study of 246 culture-confirmed melioidosis cases in Queen Elizabeth Hospital, Sabah, Malaysia was performed between 2016 and 2018. The epidemiological data and clinical and laboratory findings were extracted and analysed.

**Results:**

The annual incidence of culture-confirmed melioidosis cases was estimated to be 4.97 per 100,000 people. The mean age of the patients was 50±15 years. Males and members of the Kadazan-Dusun ethnic group accounted for the majority of the melioidosis cases. The odds ratio analysis indicated that bacteraemic melioidosis in this region was significantly associated with fever (76%), and patients having at least one underlying illness (43%), including diabetes mellitus (32%). Sixty-eight patients (28%) succumbed to melioidosis. Contrary to what is known regarding factors that promote bacteraemic melioidosis, neither patients with fever nor patients with at least one comorbid disease, including diabetes mellitus, were significantly associated with death from melioidosis. There was no statistically significant difference between patients without comorbidities (24, 27%) and those with at least one comorbid disease (26, 25%), including diabetes mellitus (18, 23%). The odds ratios indicate that melioidosis mortality in this region is related to patients showing respiratory organ-associated symptoms (29%), bacteraemia (30%), and septic shock (47%). *Burkholderia pseudomallei* isolates in this study were highly susceptible to ceftazidime (100%), imipenem (100%), and trimethoprim-sulfamethoxazole (98%).

**Conclusions:**

Information obtained from this study can be used by clinicians to recognise individuals with the highest risk of acquiring melioidosis, estimate an accurate prognosis, and provide effective treatment for melioidosis patients to reduce death from melioidosis.

## Author summary

Melioidosis is an infection caused by the bacteria *Burkholderia pseudomallei* bacterium and is most commonly found in tropical regions such as northern Australia and Southeast Asia, including Sabah, Malaysia. Death due to the disease is often associated with failure in clinical identification, prediction of death, and treatment of the disease. A local database on melioidosis could assist doctors, in particular infectious disease physicians, in overcoming these failures. Doctors in Sabah should have a high clinical suspicion of melioidosis, particularly for male patients, and particularly those of the Kadazan-Dusun ethnic group, who present with fever and have at least one underlying disease, particularly diabetes. Patients with blood infections who develop septic shock or have affected respiratory organs should be prioritised during treatment and management due to their poor prognosis. Melioidosis patients with or without an existing illness, particularly diabetes, should also be equally prioritised to prevent death. In this region, ceftazidime, imipenem, and trimethoprim-sulfamethoxazole remain highly effective antibiotics against *B*. *pseudomallei*. This new knowledge may contribute to better melioidosis clinical recognition, prognosis, and treatment of the disease to reduce mortality rates due to melioidosis.

## Introduction

Up to 165,000 melioidosis cases are estimated to occur worldwide every year [1]. However, it is believed that this number is an underestimate as many cases of melioidosis go unreported, most likely due to difficulties in clinical and laboratory diagnosis. Sepsis associated with melioidosis is often fatal, particularly in infants and immunocompromised individuals [2,3]. Limmathurotsakul et al. [1] estimated that global mortality due to melioidosis is higher than other infectious diseases such as leptospirosis and dengue, but these high mortality rates receive little attention from both local media and local authorities. Delays in treatment due to inaccurate clinical diagnosis are one of the leading causes of the high mortality rates reported in some countries [4]. Since the introduction of antibiotics to treat pathogenic bacteria, the earliest record showed that the melioidosis mortality rate in Malaysia was 65% [5], and it is suggested that these rates have not changed over the years.

The clinical presentation of melioidosis varies depending on the age of the patients, immunity levels, or complications resulting from underlying diseases such as diabetes mellitus (DM), chronic kidney disease (CKD), thalassemia, and immunodeficiency [4,6]. Although the signs and symptoms of the disease are commonly reported as fever, headache, respiratory distress, abdominal discomfort, joint pain, and/or disorientation [6,7], other unique symptoms have also been reported, particularly among children in certain regions, such as acute suppurative parotitis in North East Thailand [8,9], and Cambodia [10] or lacrimal gland infection in Sarawak, Malaysia [11]. Similarly, in terms of associated risk factors, kava consumption has only been reported in Darwin, Australia [12,13,14]. The diversity in melioidosis clinical presentations and risk factors, and the urgent need for more accurate and effective clinical recognition, has prompted the need for regional epidemiological and clinical spectrum data extraction and analyses. It is anticipated that the availability of specific regional data would assist clinicians during differential diagnosis.

The current treatment protocol for melioidosis is divided into an acute phase and an eradication phase [15]. The purpose of the acute phase treatment is to prevent patients from dying from disseminated sepsis, whereas the eradication phase is aimed at killing residual bacteria and preventing relapse or re-infection. Ceftazidime (CAZ) and carbapenems (for severe infection) are the main antibiotics administered during the acute phase. Trimethoprim-sulfamethoxazole (SXT) or amoxicillin-clavulanic acid (AMC), on the other hand, are recommended to eradicate the pathogen. CAZ, SXT, and carbapenems have been reported to reduce mortality by 50%, as most clinical isolates of *B*. *pseudomallei* are highly susceptible to these antibiotics under *in vitro* conditions [16,17]. Although relatively rare, *B*. *pseudomallei* resistance to antibiotics can emerge during treatment due to the expression of chromosomally encoded resistance determinants [18,19]. Resistance may become more prevalent in regions where melioidosis is endemic. Therefore, periodic surveillance of antibiotic susceptibility patterns is essential to assist clinicians in determining the most effective antibiotics against local strains of *B*. *pseudomallei*.

Over the last few decades, a number of epidemiological studies have been reported showing demographics, clinical spectrum, prognosis, and laboratory and antibiotic susceptibility testing (AST) findings in melioidosis patients to facilitate accurate clinical diagnosis and treatment. However, the information is restricted to specific locations within Malaysia [5,11,20,21,22,23,24]. Sabah is one of 13 states in Malaysia and is located on the island of Borneo. In Sabah, the first melioidosis case report was published in 1978 [25]. Later, a brief epidemiology-related report was published in 2014 [26], revealing a high prevalence of melioidosis cases among individuals aged 40 to 60 years engaged in work associated with the agriculture sector. The latest Sabah epidemiology study on paediatric melioidosis patients was published in 2015 [3]. Although the Sabah State Health Department requires that melioidosis cases are notified administratively, it is suspected that under-reporting is still a major problem, similar to all other states in Malaysia [4,27].

Under-reporting of melioidosis cases in Sabah has resulted in a dearth of local epidemiological and treatment information, leading to difficulties in clinical recognition, prognosis, and appropriate treatment of patients. The rapid urbanisation that has taken place in Kota Kinabalu, the capital of the state of Sabah, over the last couple of decades has led to landscape changes, a factor that is closely associated with increases in the number of melioidosis cases [28]. Taken together, the lack of melioidosis data and the rapid urbanisation of Sabah prompted this retrospective study to be performed. The insights from this study are expected to improve clinical identification, prognosis, and treatment of melioidosis patients, thereby lowering the mortality rates. Here we undertook a three-year retrospective study based on an examination of the demographics, clinical spectrum, prognosis, and treatment of the disease. Relevant statistical analyses were used to confirm the demographics data, clinical spectrum, and efficacy of the recommended treatment.

## Methods

### Ethics statement

Ethical approval to conduct the study was obtained from the Medical Research and Ethics Committee (MREC), Ministry of Health, Malaysia (NMRR-18-3713-45583 IIR). All data were derived from retrospective and secondary data. Ethical approval included access to the 246 isolates data available at the Queen Elizabeth Hospital (QEH).

### Study site and population

A retrospective study of culture-confirmed melioidosis patients was conducted at the QEH, a 779-bed tertiary hospital located in Kota Kinabalu, Sabah, Malaysia. Melioidosis is an administratively notifiable disease in Sabah, thus all microbiological specimens of clinically diagnosed suspected cases from the Sabah west coast region peripheral health clinics and district hospitals (including Ranau, Kota Belud, Tuaran, Penampang, Papar, Kudat, Kota Marudu, Pitas, Beaufort, Kuala Penyu, Sipitang, and Putatan) (S1 Fig) are referred to QEH for further case confirmation and/or patient treatment and management. We retrospectively identified culture-positive cases referred to QEH from January 2016 to November 2018. Records were retrieved from laboratory requisition forms and laboratory databases and entered into a standard electronic collection form, analysed and reviewed.

Demographic data (gender, age, ethnicity, and subsequent finding/outcome) and clinical spectrum data (signs, symptoms, and risk factors) were extracted from laboratory requisition forms, while antibiotic susceptibility patterns were obtained from laboratory database. Requisition forms with insufficient clinical summaries were classified as unknown and were excluded from the data calculation and statistical analysis.

Data for 2018 on the total population of Kota Kinabalu and the nearby districts, Sabah’s average gender ratio from 2016 to 2018, and Sabah’s average ethnicity proportion from 2016 to 2018 were obtained from the Department of Statistics, Malaysia. The reported Kota Kinabalu and nearby districts’ population for 2018 and the numbers of cases averaged over the study period of 2016 to 2018 were used to calculate the annual incidence (per 100,000 population). The ethnicity of melioidosis patients was classified as Malay, Chinese, Indian, Kadazan-Dusun, other indigenous ethnic, Bajau, Murut, or non-Malaysian. The antibiotic upper susceptibility limits were extracted from the Clinical and Laboratory Standard Institutes (CLSI) guidelines [29]. The ages of the patients were classified based on the World Health Organisation Guidelines [30,31].

### Climatic factor

To determine any potential association between cases and environmental factors, the months when the melioidosis patients were admitted to QEH were recorded and compared with the average monthly rainfall data for Kota Kinabalu from 2016 to 2018. The average monthly rainfall data for Kota Kinabalu from 2016 to 2018 was obtained from the Malaysian Meteorological Department.

### Definitions

Patients were considered bacteraemic if blood cultures were positive, and non-bacteraemic if cultures were only positive from other sources (sputum, pus, urine, etc). Septic shock refers to the circulatory failure caused by persistent arterial hypotension (systolic arterial pressure <90 mmHg with a mean arterial pressure lower than 60 or a reduction in systolic arterial pressure of >40 mmHg from baseline, despite adequate volume resuscitation) [32]. The urban area was defined as areas in the city of Kota Kinabalu itself, whereas the sub-urban areas were defined as districts outside Kota Kinabalu. Risk factors such as DM, hypertension, pulmonary tuberculosis (PTB), CKD, Human Immunodeficiency Virus (HIV) infection, thalassemia, hepatitis infection, and malignancy were identified based on documentation in the medical records. Symptoms were classified based on the organ(s) affected as shown in S1 Table.

### Microbiological methods

The study included all patients admitted to QEH and those that were referred to QEH from neighbouring hospitals in the region (S1 Fig). The presence of the bacteria was confirmed microbiologically at QEH and was positive in at least one specimen (blood, sputum, urine, tissue, pus, or body fluids) (*n* = 246). Briefly, blood specimens were first incubated in a commercial BacT/ALERT culture medium (bioMérieux, France) in the BacT/ALERT 3D automated microbial detection system (bioMérieux, France). Positive cultures from the BacT/ALERT bottle were subcultured on blood agar and MacConkey’s agar. Specimens from other sources were cultured directly on blood agar and MacConkey’s agar and incubated at 37°C. Suspected *B*. *pseudomallei* isolates were subjected to further tests such as Gram stain and the ability to grow at 42°C, as initial tests to identify *B*. *pseudomallei*. Species confirmation was further obtained from the automated VITEK 2 compact system (bioMérieux, France) and/or the Remel RapID NF Plus kit according to the respective manufacturer’s instructions [33,34,35].

AST was determined using the gradient Minimum Inhibitory Concentration (MIC) strip method [36]. The MIC breakpoint and interpretation standards for susceptible (S), intermediate (I), and resistant (R) were carried out following the guidelines published by CLSI [29]. The antibiotics tested were CAZ, imipenem (IPM), SXT, AMC, and tetracycline (TET). The *Escherichia coli* ATCC 25922 isolate was used as the control for all the antimicrobial MIC susceptibility tests.

### Statistical analysis

A database was built and statistical calculations were performed using IBM SPSS Statistics version 26.0 (IBM Corp., USA). Descriptive analysis was presented by percentage data according to each category, and the mean and standard deviation were calculated for continuous variables.

The binomial test was used to test if an association existed between the melioidosis patients’ gender proportion and population proportion. The Pearson χ2 test or Fisher’s exact test was used to determine the association of different variables with bacteraemic melioidosis. Odds ratios (ORs) were used to assess the strength of the association between variables and both bacteraemic melioidosis and melioidosis mortality. The relationship between the total number of melioidosis cases and the average monthly rainfall was estimated using Pearson or Spearman correlation. To calculate the significance value of the difference between the observed and expected melioidosis patients based on population ethnic proportions, the Exact Monte Carlo Pearson’s Chi-Square Goodness-of-Fit test was used. The One-Sample Median Test (Wilcoxon Signed Rank Test) was performed to determine the difference between observed and hypothesised median MIC values, respectively. A *p*-value of <0.05 was considered significant.

## Results

### Demographic data

Demographic data, symptoms, and existing underlying disease are presented in Table 1. A total of 246 melioidosis cases were recorded at QEH during the three-year study period. The calculated annual incidence of melioidosis was 4.97 per 100,000 people. Overall, males were noted to have a higher risk of melioidosis (*n* = 188, 76%). A binomial test revealed that the male to female ratio in this study (0.76) was significantly greater than the average local population’s ratio of males to females (0.51) (*p* <0.001) as determined from the Department of Statistics census records. The ages of all the melioidosis patients ranged from 7 months to 85 years (mean: 50 ± 15 years; median: 52 years), with females slightly older than males (Table 1). The majority of the cases occurred in patients aged between 45 and 64 years (*n* = 123, 50%), followed by the age group of 25 to 44 years (*n* = 70, 28%). Interestingly, most of the children and infants aged less than 15 years old had a bacteraemic infection, including a 7-month-old female infant (age group: <1 year) and a 1-year-old male who developed fever and severe respiratory distress and later succumbed to the infection.

**Table 1:** Epidemiology of culture-confirmed melioidosis patients in QEH between January 2016 and November 2018.

	Melioidosis cases (*n* = 246)	Bacteraemia	Non-Bacteraemia	Pearson/Fisher
				*p-*value ^b^
**Male to Female Proportion**	188:58 (76%:24%)			
Male	188 (76%)	173	15	Binomial test <0.001
Female	58 (24%)	54	4	
**Mean age (years)**				
Male	50 ± 14			
Female	50 ± 18			
Total	50 ± 15			
**Median age** **years (interquartile range)**				
Male	52 (IQR: 41–60)			
Female	55 (IQR: 40–63)			
Total	52 (IQR: 41–61)			
**Age group (years)** ^**a**^				
<1	1 (0%)	1	0	1.00
1–14	7 (3%)	5	2	0.09
15–24	6 (2%)	5	1	0.39
25–44	70 (28%)	65	5	0.83
45–64	123 (50%)	115	8	0.47
65+	37 (15%)	34	3	1.00
Unknown	2 (1%)	2	0	1.00
**Residential area**				
Urban	158 (64%)	145	13	0.69
Sub-urban	88 (36%)	82	6	
**Ethnicity**				
Malay	20 (8%)	19	1	Pearson’s chi-square goodness-of-fit <0.001
Chinese	35 (14%)	32	3	
Indian	1 (0%)	0	1	
Kadazan-Dusun	83 (34%)	77	6	
Other indigenous ethnic groups	43 (17%)	40	3	
Bajau	37 (15%)	35	2	
Murut	7 (3%)	5	2	
Non-Malaysians	20 (8%)	19	1	

^a^ Based on the requisition form completed by the clinicians. A characteristic was labelled as unknown if the information was missing from the requisition forms.

^b^ A p-value <0.05 was considered statistically significant.

**Fig 1:**
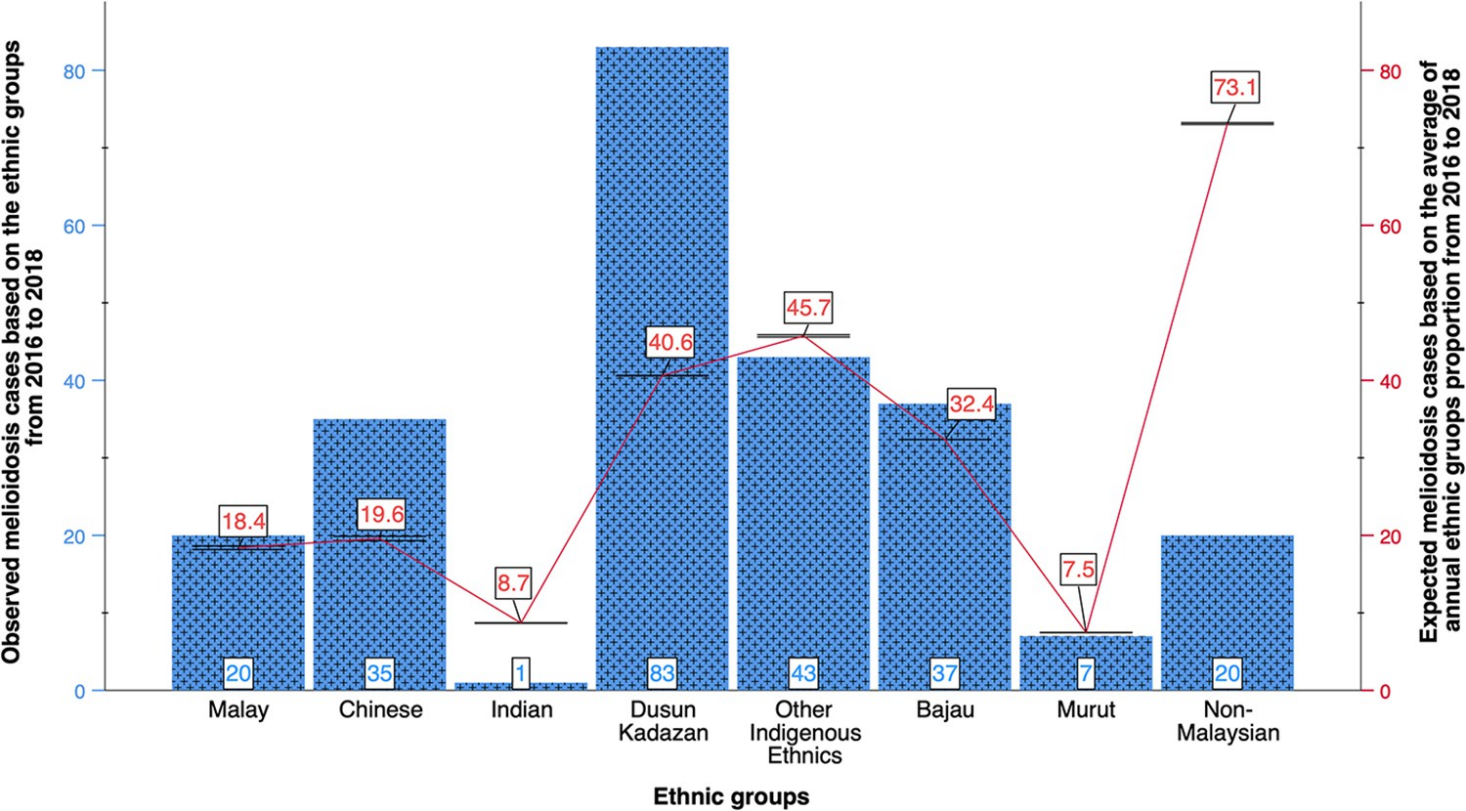
Distribution of the 246 culture-confirmed melioidosis cases based on ethnic groups sampled during the study and the expected melioidosis cases based on the annual average proportion of the Sabah ethnic groups over the 3 years period (January 2016—December 2018) in Sabah. (A) The bar chart (blue) shows the distribution of the 246 melioidosis cases according to the ethnic groups. (B) The expected melioidosis cases based on the average annual ethnic groups proportion is shown in the line graph (red).

The majority of the patients lived in urban areas (64%). Most patients were of the Kadazan-Dusun ethnic group (34%), other indigenous ethnic groups (17%), Chinese (14%) and Bajau (15%) ethnic groups, with other minorities (20%) making up the remainder (Table 1). The Exact Monte Carlo Pearson’s Chi-Square Goodness-of-Fit test revealed that there are statistically significant differences in the ethnic proportions of melioidosis patients in this study compared to the average general ethnic proportions (χ^**2**^ = 79.29, df = 7, *p* <0.001). The number of infection cases among the Indian (*n* = 1) and non-Malaysians (*n* = 7) populations was less than expected, whereas the number among Chinese and Kadazan-Dusun patients was more than anticipated (Fig 1).

Bacteraemic melioidosis was seen in the majority of patients (227, 92%). Nonetheless, there was no significant association between residential area and bacteraemic melioidosis in this population (*p* = 0.69).

### Common symptoms and risk factors of melioidosis patients

Clinical spectrum data were available for 194 patients with culture-confirmed melioidosis. Fever was the most common symptom recorded, accounting for 76% of all confirmed melioidosis cases (*n* = 186, *p* <0.001).

While there were no statistically significant relationships between bacteraemic melioidosis and affected respiratory, cardiovascular, musculoskeletal, nervous, genitourinary, lymphatic, or digestive organ sites, respectively (*p* ≥0.05), several statistically significant associations were observed, including between bacteraemic melioidosis and fever (*p* = 0.02), and bacteraemic melioidosis and symptoms within the integumentary organ system (*p* <0.001) (Table 2). The ORs supported these findings for fever (OR: 7.99, 95% CI: 1.72–37.18) and symptoms observed for the integumentary organ system (OR: 0.03, 95% CI: 0.01–0.11). Taken together, patients who presented with fever were 7.99 times more likely to develop bacteraemic melioidosis than those who did not present with fever. In contrast, the odds of a patient with integumentary organ system symptoms developing bacteraemic melioidosis was 97% lower than in patients with non-integumentary symptoms, such as fever or an affected respiratory organ.

**Table 2:** Sign/Symptoms, underlying illnesses, general health status and outcome of culture-positive melioidosis patients in QEH between January 2016 and November 2018.

	Melioidosis cases (*n* = 246)	Bacteraemia	Non-Bacteraemia	Pearson/Fisher ^c^	Odds ratio ^d^	
				*p-*value ^e^	OR	95% CI
**Signs/** **Symptoms** ^a^^,^^b^						
Fever	186 (76%)	173	13	0.02	7.99*	1.72–37.18
Cardiovascular	4 (2%)	4	0	1.00		
Musculoskeletal	22 (9%)	21	1	1.00		
Integumentary	27 (11%)	15	12	<0.001	0.03*	0.01–0.11
Respiratory	160 (65%)	148	12	0.49		
Nervous	7 (3%)	7	0	1.00		
Genitourinary	19 (8%)	18	1	1.00		
Lymphatic	4 (2%)	3	1	0.29		
Digestive	47 (19%)	46	1	0.12		
Unknown	52 (21%)	49	3	0.77		
**Underlying illnesses** ^a^^,^ ^b^						
DM	79 (32%)	77	2	0.02	5.34*	1.18–24.18
Hypertension	50 (20%)	49	1	0.08		
CKD	26 (11%)	26	0	0.14		
Cardiovascular diseases	15 (6%)	15	0	0.62		
Thalassemia major	2 (1%)	2	0	1.00		
Malignancy	3 (1%)	3	0	1.00		
PTB	7 (3%)	6	1	0.46		
HIV	1 (0%)	1	0	1.00		
Hepatitis infection	2 (1%)	2	0	1.00		
Leptospirosis	3 (1%)	3	0	1.00		
**General health status**						
Had at least one underlying illness	106 (43%)	103	3	0.01	5.95*	1.64–21.62
No underlying illness	88 (36%)	75	13	0.00	0.17*	0.05–0.61
Unknown	52 (21%)	49	3	0.77		
Total	246 (100%)	227	19	-		
**Patients’ outcome**						
Septic shock	55 (22%)	54	1	0.04	6.53	0.84–50.71

OR, odds ratio; CI, confidence interval.

^a^ Based on the requisition form completed by the clinicians. A characteristic was labelled as unknown if the information was missing from the requisition forms.

^b^ Some patients may have had more than one symptom and/or underlying illness.

^c^ To test the association between symptoms/underlying illnesses and bacteraemic/non bacteraemic melioidosis.

^d^ To measure the strength of association between symptoms/underlying illnesses and bacteraemic melioidosis.

^e^ A p-value <0.05 was considered statistically significant.

* Indicates a *p*-value <0.05 (statistically significant)

Interestingly, almost half (*n* = 106, 43%) of the melioidosis patients in this study had at least one underlying illness, with DM as the most common underlying illness or comorbidity (*n* = 79, 75%). Among the rest of the patients (*n* = 140, 57%), 88 patients had no underlying illness (63%) and 52 patients had an unknown clinical history (37%). Significant associations were observed between bacteraemic melioidosis and patients with at least one underlying disease (χ^**2**^ = 6.25, df = 1, *p* = 0.01) (OR: 5.95, 95% CI: 1.64–21.62) and those with DM (χ2 = 5.75, df = 1, *p* = 0.02) (OR: 5.34, 95% CI: 1.18–24.18). Hence, the odds of developing bacteraemic melioidosis were 5.95 times higher in patients who had at least one underlying illness compared to patients with no underlying illness, and the odds of developing bacteraemic melioidosis were 5.34 times higher in diabetics compared to non-diabetics.

As expected, there was a significant negative association observed between bacteraemic melioidosis and patients with no underlying illness (χ2 = 9.55, df = 1, *p* <0.01), which was further corroborated by the OR test (OR: 0.17, CI 95%: 0.05–0.61). Therefore, patients who did not have any underlying illness were 83% less likely to get bacteraemic melioidosis than those who did have an underlying illness. A significant association between bacteraemic melioidosis (*n* = 54) and septic shock (*n* = 55) as a subsequent outcome was noted (*p* = 0.04). Nonetheless, the calculated odds ratio did not support our observation (OR: 6.53, 95% CI: 0.84–50.71).

### No correlation between the number of melioidosis cases and monthly rainfall

**Fig 2:**
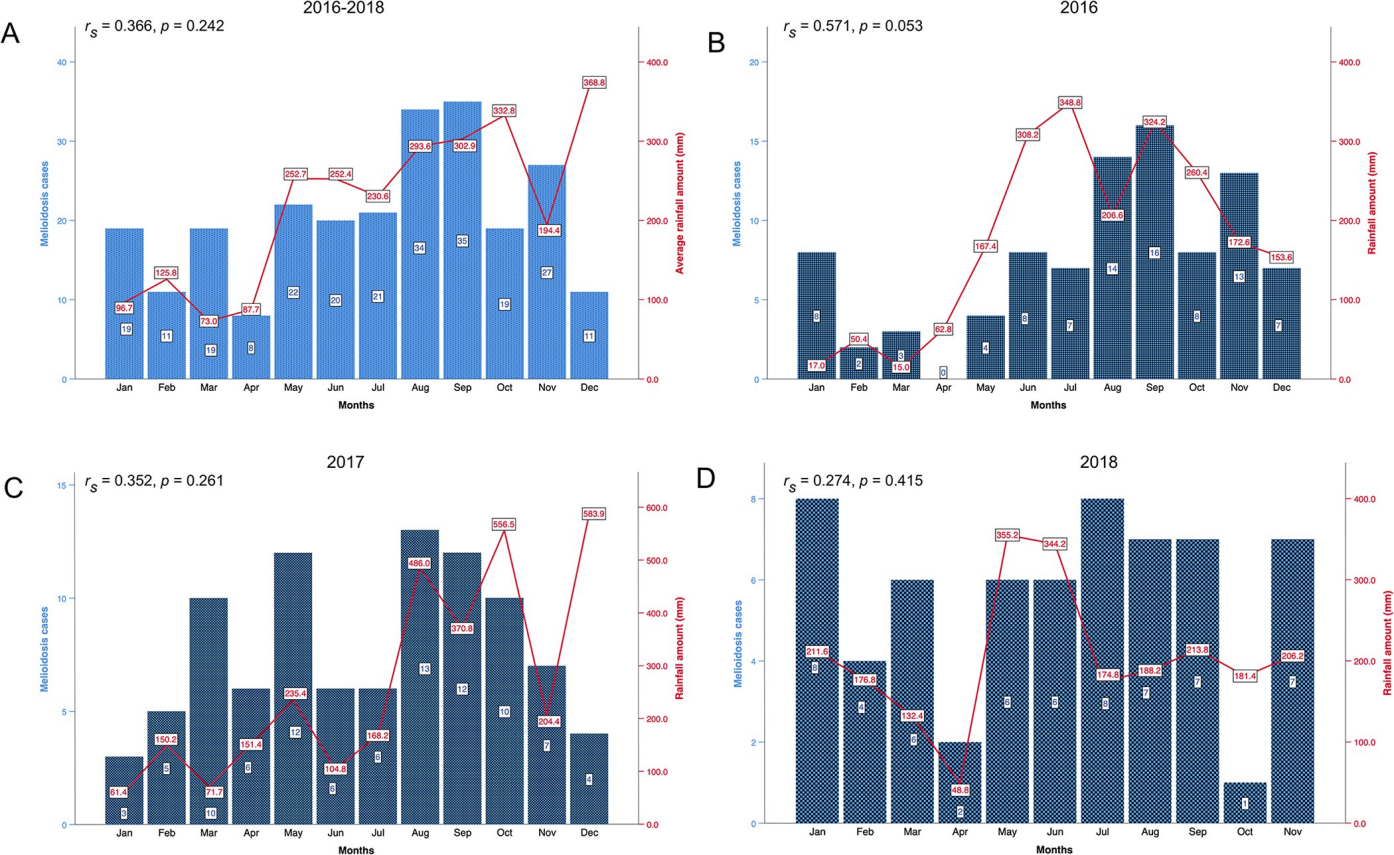
Distribution of the 246 culture-confirmed melioidosis cases based on the monthly rainfall. (A) Distribution of the 246 melioidosis cases and average monthly rainfall over the 3 year period (January 2016—November 2018) (*r*_*s*_ = 0.366, *p* = 0.242). (B) Distribution of the 90 melioidosis cases and monthly rainfall for 2016 (*r*_*s*_ = 0.571, *p* = 0.053). (C) Distribution of the 94 melioidosis cases and monthly rainfall for 2017 (*r*_*s*_ = 0.352, *p* = 0.261). (D) Distribution of the 62 cases and monthly rainfall for 2018 (*r*_*s*_ = 0.274, *p* = 0.415). The bar chart depicts the distribution of melioidosis cases according to the month of admission. The corresponding average monthly rainfall amount (A) and monthly rainfall (B, C & D) in the vicinity of Kota Kinabalu is shown by the line graph.

Fig 2 depicts the association between the occurrence of melioidosis cases in relation to the observed mean monthly rainfall in the vicinity of Kota Kinabalu region. More than half of the cases (*n* = 132, 54%) occurred during the wet season and increased linearly with the mean monthly rainfall received from the months of May to September in the three-year study period (Fig 2A). However, there was no statistically significant correlation between the number of melioidosis cases and monthly rainfall observed (*r*_*s*_ = 0.366, *p* = 0.242). Similar findings were observed for each of the individual years (2016; *r*_*s*_ = 0.571, *p* = 0.053, 2017: *r*_*s*_ = 0.352, *p* = 0.261 and 2018: *r*_*s*_ = 0.274, *p* = 0.415) (Fig 2A–2C).

### Case fatality

The case-fatality ratio among culture-confirmed melioidosis cases was 28% (S2 Table). Based on the study population, a poor prognosis is predicted for those who have bacteraemia, septic shock, or respiratory organ systems failure. Patients with bacteraemia have a 7.54-fold higher odds of dying than non-bacteraemic patients. Patients with septic shock are 4.3 times more likely to die than patients not presenting with septic shock. In patients with compromised respiratory organs, a similar outcome has been observed. Patients with affected respiratory organs are 4.3 times more likely to die than those whose respiratory organs were not affected. The majority of deaths (*n* = 48, 71%) were observed in patients over the age of 45.

It is interesting to note that the mortality rate of melioidosis patients without an underlying illness (*n* = 24, 27%) was not significantly different to the mortality rate attained by patients with at least one underlying illness (*n* = 26, 25%), particularly patients with DM (*n* = 18, 23%). This finding indicates that the risk of contracting melioidosis is independent of the risk of death for these patient groups.

### The recommended antibiotic treatment panel is relevant and effective against local *B*. *pseudomallei* isolates

*B*. *pseudomallei* isolates from 244 melioidosis patients were tested for AST, and the results in this population indicated that the CLSI-recommended antibiotic panel is still effective for the treatment of melioidosis. Overall, all antibiotics tested were effective against the local *B*. *pseudomallei* isolates. All *B*. *pseudomallei* isolates in this study were susceptible to CAZ (*n =* 244/244, 100%) and IPM (*n =* 210/210, 100%), followed by SXT (*n =* 236/242, 98%) and AMC (*n* = 186/204, 91%). The lowest susceptibility was observed for TET (*n* = 188/220, 85%).

The antibiotic MIC median values were significantly lower than their respective susceptibility limit values, including CAZ (median = 1.0, IQR: 0.8–1.0), IPM (median = 0.4, IQR: 0.3–0.4), SXT (median = 0.3, IQR: 0.1–0.8), AMC (median: 3.0, IQR: 1.5–4.0), and TET (median: 3.0, IQR: 2.0–4.0) (Table 3).

**Table 3:** Antibiotic MIC median values (μg/mL) against local *B*. *pseudomallei* isolates in comparison with their respective susceptibility upper limit values.

Antibiotic	*n* ^a^	Median MIC (μg/mL)	IQR	Susceptibility upper limit values (μg/mL) ^b^	*p*-value ^c^
SXT	242	0.3	0.1–0.8	2.0	<0.001
AMC	204	3.0	1.5–4.0	8.0	<0.001
CAZ	244	1.0	0.8–1.0	8.0	<0.001
IPM	210	0.4	0.3–0.4	4.0	<0.001
TET	220	3.0	2.0–4.0	4.0	<0.001

MIC, minimum inhibitory concentration; SXT, trimethoprim-sulfamethoxazole; AMC, amoxicillin/clavulanic acid; CAZ, ceftazidime; IPM, imipenem; TET, tetracycline; IQR, interquartile range

^a^ Specimens with no MIC results were classified as ‘unknown’ and excluded from the calculation and statistical analysis.

^b^ Based on the CLSI Guidelines [29].

^c^ *p*-value <0.05 showed that a significant difference in value was observed between the tested antibiotic.

## Discussion

The main findings of this retrospective study on melioidosis are the high proportion of bacteraemia occurrences and the high incidence rate, which are causes for concern. In this study, bacteraemic melioidosis was significantly linked to patients who had fever, at least one underlying illness, and/or DM. However, contrary to expectations, no specific patient group was significantly associated with a higher risk of mortality. In fact, there was no significant difference between the mortality rates of patients with no underlying illness and those with at least one underlying illness, particularly DM.

The occurrence of melioidosis did not appear to be statistically significant in the population tested in relation to rainfall, despite the fact that more than half of the melioidosis cases occurred during the wet season. Research findings from South East Asia [7,16,20,37,38], East Asia [39,40], and Australia [12,41] have previously indicated that high rainfall correlates with an increase in melioidosis cases. In contrast, several centres, including Kuala Lumpur and Sarawak in Malaysia [11,42], Thailand [43], and Singapore [44], have reported no significant correlation. As we could not establish a relationship between the sudden increase in melioidosis cases and excessive rainfall in Sabah, we hypothesise that the increase seen in this study may be attributable to other factors, such as humidity levels [38] or irregular rainfall patterns across the catchment areas [42].

Although almost half (*n* = 106, 43%) of the melioidosis patients in this study had at least one underlying comorbidity, we believe that this was an underestimate. The number of cases recorded with an "unknown" medical history (*n* = 52, 21%) may have contributed to the underestimation. Meanwhile, about one-third (*n* = 88) of the melioidosis patients had no underlying illness, justifying the findings by Toh et al. [24]. This suggests that environmental factor(s) might play a role in the incidence of melioidosis within our locality.

In this study, the annual melioidosis incidence rates were higher than the calculated incidence in urban cities such as Kuala Lumpur [5] and Singapore [44,45]. As there was no correlation between rainfall and disease incidence observed in these cities [42,44] and the significant increase in melioidosis cases compared to those reported between 2011 and 2013 [26], we postulate that the higher incidence in the Kota Kinabalu area could be due to the rapid and active urbanisation of the city, particularly the construction and excavation activities during the development of the Pan Borneo Highway [46]. Urbanisation and new highway construction were also reported to contribute to an increase in melioidosis cases in Queensland, Australia [47]. Furthermore, QEH also receives patients and conducts confirmatory culture tests from neighbouring districts, where most of the population is still dependent on the agricultural sector, which has been reported to be significantly associated with melioidosis [46,48,49].

Elderly patients over the age of 45 years accounted for more than half of the confirmed melioidosis cases and melioidosis mortality, confirming previous reports that highlighted the high incidence of melioidosis cases and mortality among people of a similar age group [4,7,50]. This phenomenon has been attributed to the increased susceptibility to *B*. *pseudomallei* infection for individuals with comorbid diseases [6].

Most infants and children in this study had bacteraemia (6/8, 75%), which consolidated previous study findings emphasising the high proportion of bacteraemic melioidosis in children and infants in the Borneo region, with 74% (20/27) [3] and 55% (23/42) [11] of the total confirmed melioidosis cases. Similar proportion have been observed throughout Malaysia at a rate ranging from 44% to 93%) [6]. The results of our study differ from previous research from other locations, which frequently highlighted localised cutaneous infection as a primary symptom in paediatric melioidosis, such as acute suppurative parotitis commonly observed in North East Thailand [8,9], and Cambodia [10]. The observed discrepancy may be attributed to potential factors such as (1) the actual situation in which there were higher ratios of bacteraemic patients as compared to non-bacteraemic patients, in contrast to the reports elsewhere, (2) misdiagnosis of melioidosis parotitis as a viral infection, thus no pus specimen was taken for culture, (3) failure to set up cultures from pus or wound samples, and (4) unawareness of melioidosis among parents who then failed to send their children to the hospital, particularly those with mild infections and symptoms such as parotitis or lacrimal gland infections. A similar postulation has been made for the higher bacteraemic melioidosis cases observed in adult patients as compared to the studies elsewhere. Therefore, a further study incorporating prospective data related to signs and symptoms should be undertaken to confirm the hypothesis.

This study also demonstrated that males were more likely than females to contract a *B*. *pseudomallei* infection and present with melioidosis symptoms, supporting previous observations from other Malaysian states, including Kelantan [7], Kedah [20], Pahang [21,51], Kuala Lumpur [5, 51] and Perak [22], of increased association between males and the disease. Similar findings have been reported in other countries such as Singapore [16,45], northern Australia [52], Hong Kong [50], Cambodia [37] and Thailand [43,53,54].

In this study, fever was the most typical symptom of melioidosis recorded in the requisition form. Our results reinforce the reports from other states in Malaysia and across the Southeast Asia region that bacteraemic melioidosis is commonly observed in patients with fever [7,16,43]. On the contrary, patients with an affected integumentary organ system are unlikely to have bacteraemic melioidosis, in which a negative result for blood culture in these patients is expected.

Patients in our population with at least one underlying illness and DM were significantly more likely to have bacteraemic melioidosis; these results corroborate previous conclusions [4,6,50] that the elevated disease prevalence observed for DM patients and patients who had at least one underlying illness in our population is due to a compromised immune system [55,56].

Kadazan-Dusun and Chinese ethnic groups contributed to the highest number of melioidosis cases, significantly higher than the expected cases based on the population ratio. While this unexpected observation may be attributed to differences in exposure rates and types of occupation [6,51], the exact mechanism by which they acquired the infection is still unknown. In the future, patients occupation records would be central to confirming this observation.

Melioidosis is an administratively notifiable disease in the Sabah region and a local clinical practice guideline outlining the recommended standard antimicrobial treatment is available in all hospitals [26]. Nonetheless, our study revealed a mortality rate of 28% among melioidosis patients thus affirming the severity of the disease. Higher mortality rates were reported in Thailand [54], Taiwan [39], and Cambodia [37]. In contrast, lower mortality rates of 14% and 21% were reported in Australia [12,57]. In Malaysia, various mortality rates have been reported in different states, ranging from 12% to 65% [3,5,7,11,20,21,22,23,24,51]. Recently, the lowest mortality rate (6%) ever documented was recorded in Darwin, Australia in a 30-year study period due to the application of an intensivist-led model of care and the empirical prescription of meropenem for critically ill patients [58]. The significant mortality rate observed in our study suggests that treatment of melioidosis patients may be more difficult if the infection has progressed, and treatment may also be impeded by underlying comorbidity factors.

Other factors that may have contributed to the mortality rates observed in this study include differences in *B*. *pseudomallei* virulence based on geographical area, patient genetics and differences in the patient-pathogen response to the recommended therapy [20]. An important point of note is the high proportion of patients with bacteraemia at the time of hospital admission, which is similar to the rate previously reported in Perak, Malaysia [22]. Unfortunately, when the bacteria is present in the blood, treatment is less effective, which may lead to septic shock and eventually death. The risk of the disease progressing from severe sepsis to septic shock has been shown to be significantly associated with a delay in administering the recommended antimicrobials [59]. Therefore, it is imperative to implement a proactive plan that adopts the approach employed in Darwin, Australia [58] to reduce mortality rates.

In this study, bacteraemia, septic shock, and the affected respiratory organs (pulmonary involvement) were shown to be significant predictors of mortality. Similar results were observed in previous studies carried out in Malaysia [11,22] and the Southeast Asia region [16,43,60]. Patients with bacteraemia had significantly more severe sepsis than non-bacteraemic patients [61], which explains why the majority of patients with septic shock outcomes in this study (*n* = 55) were also bacteraemic patients (*n =* 54), and both were significantly related to mortality. Therefore, clinicians should be made aware of the significant potential for bacteraemic patients to develop severe sepsis to enable better patient care and outcomes. Mortality rates for the patients in this study were noted to increase in tandem with the age of the patient. This is not surprising as previous studies had also noted that increasing age was significantly associated with mortality where patients aged 65 years or older had more severely affected respiratory organ systems such as bacterial pneumonia [62,63].

We also found that the fatal outcome of patients without comorbidity (24, 27%) was not significantly different from those who had at least one comorbid disease (26, 25%), including DM (18, 23%). In this study, both DM and having at least one comorbid disease were important risk factors for acquiring melioidosis, but neither was found to be significantly related to mortality. Similar findings have been reported in previous studies conducted in Perak, Malaysia [22], and other countries, including Singapore [16] and Thailand [43]. On the contrary, Abu Hassan et al. [64] reported that melioidosis patients with DM were responsible for the majority of fatalities. Our findings are similar to previous studies that reported that those with DM were more susceptible to melioidosis but less likely to succumb to sepsis when compared to those without DM. This difference is proposed to be due to diabetics consuming glyburide or oral angiotensin-converting enzyme (ACE) to reduce hyperinflammation, a key factor that increased the odds of dying among melioidosis patients [4,65,66]. This finding suggests that melioidosis patients without underlying illness have a similar mortality risk as those with underlying illness, particularly DM. Therefore, when it comes to the treatment and management of melioidosis patients, all patients should receive equal attention in order to prevent unnecessary fatalities.

The *B*. *pseudomallei* strains in this study were highly susceptible to CAZ and IPM, which is not surprising as to date, there have been no reports on CAZ or IPM-resistant isolates from Malaysia. The occurrence of CAZ- and carbapenem-resistant *B*. *pseudomallei* due to natural selection is extremely uncommon [67]. Therefore, the selection of these antibiotics as recommended by the guidelines to treat acute melioidosis is still relevant [15,26,41,68,69]. The antibiotic susceptibility of *B*. *pseudomallei* against SXT (98%) was similar to the reports published in Australia [70] and Kuala Lumpur, Malaysia [71] but higher compared to that reported by Ahmad et al. [72] and Arushothy et al. [73]. Nonetheless, it should be noted that the susceptibility data for SXT in the aforementioned studies may have overestimated resistance due to possible mistakes in reading the 80% endpoint, as reported by Saiprom et al. [74].

Therefore, we recommend that CAZ, IPM and SXT continue to be used as the treatment of choice for melioidosis due to the higher susceptibility percentages and their MIC values against *B*. *pseudomallei* being significantly lower than their susceptibility breakpoints. On the other hand, AMC and TET should be administered with care to avoid treatment failure, in lieu of the lower susceptibility values.

A limitation of this study is that demographic and clinical summary data were extracted from test requisition forms, which may inadvertently contain mis-represented information due to incomplete forms or misconstrued data. A prospective study incorporating data extracted from case notes and interview sessions to obtain patients’ treatment data, occupation data, and patient condition at admission, as well as the time between blood collection and release of AST results, should be integrated with the existing secondary data to improve data accuracy and enable more meaningful conclusions to be made. Furthermore, it has been reported that the use of VITEK 2 and/or Remel RapID NF Plus may lead to the misidentification of *B*. *pseudomallei* [75,76,77]. To mitigate this, *B*. *pseudomallei* isolates identified by the VITEK 2 system with average probability should be subjected to the Remel RapID NF Plus test. If a discrepancy arose between the two tests, PCR will be conducted at the reference centre.

## Conclusion

The new information given here paves the way for more efficient and accurate clinical recognition and improved prognosis and treatment of melioidosis. In Sabah, melioidosis patients commonly progress to bacteraemia and one-fourth of them eventually succumb to the infection. As such, clinicians in hospitals such as QEH should suspect melioidosis particularly for a male individual presenting with fever with at least one underlying disease, particularly DM, and who is a member of the Kadazan-Dusun ethnic group. The wet season was not an indicator of a surge in melioidosis cases in our studied population. Bacteraemic patients who develop septic shock, or with affected respiratory organ symptoms should be prioritised during treatment and management due to their poor prognosis. Melioidosis patients without existing illness and patients with at least one underlying illness, particularly DM, should be equally prioritised to prevent unnecessary death. CAZ and IPM remain highly effective for the treatment of acute melioidosis while SXT is recommended for eradication therapy.

## Supporting information

S1 Fig QEH patients and specimens coverage by districts within west coast region of Sabah. The map of Malaysia shows the Peninsular Malaysia and Malaysian states of Sarawak and Sabah in Malaysian Borneo region. The magnified view of Sabah map depicts the location of districts in Sabah (west coast region), covered by QEH with its populatio size (http://store.usgs.gov/map-locator).
(DOCX)

S1 Table Classification of the signs/symptoms based on the affected organ system. (DOCX)

S2 Table Case fatality ratio and the odds ratio for death based on demographic data, symptoms and underlying illnesses among melioidosis culture-confirmed cases. (DOCX)
