# Supplementary material for: Improving the clinical recognition, prognosis, and treatment of melioidosis through epidemiology and clinical findings: The Sabah perspective
Source: PLoS Negl Trop Dis. 2023 Oct 16;17(10):e0011696. doi: 10.1371/journal.pntd.0011696 (PMC10602235; doi:10.1371/journal.pntd.0011696)
Supplement: S1 Fig — The map of Malaysia shows the Peninsular Malaysia and Malaysian states of Sarawak and Sabah in Malaysian Borneo region. The magnified view of Sabah map depicts the location of districts in Sabah (west coast region), covered by QEH with its populatio size (http://store.usgs.gov/map-locator). (DOCX) [file pntd.0011696.s001.docx]

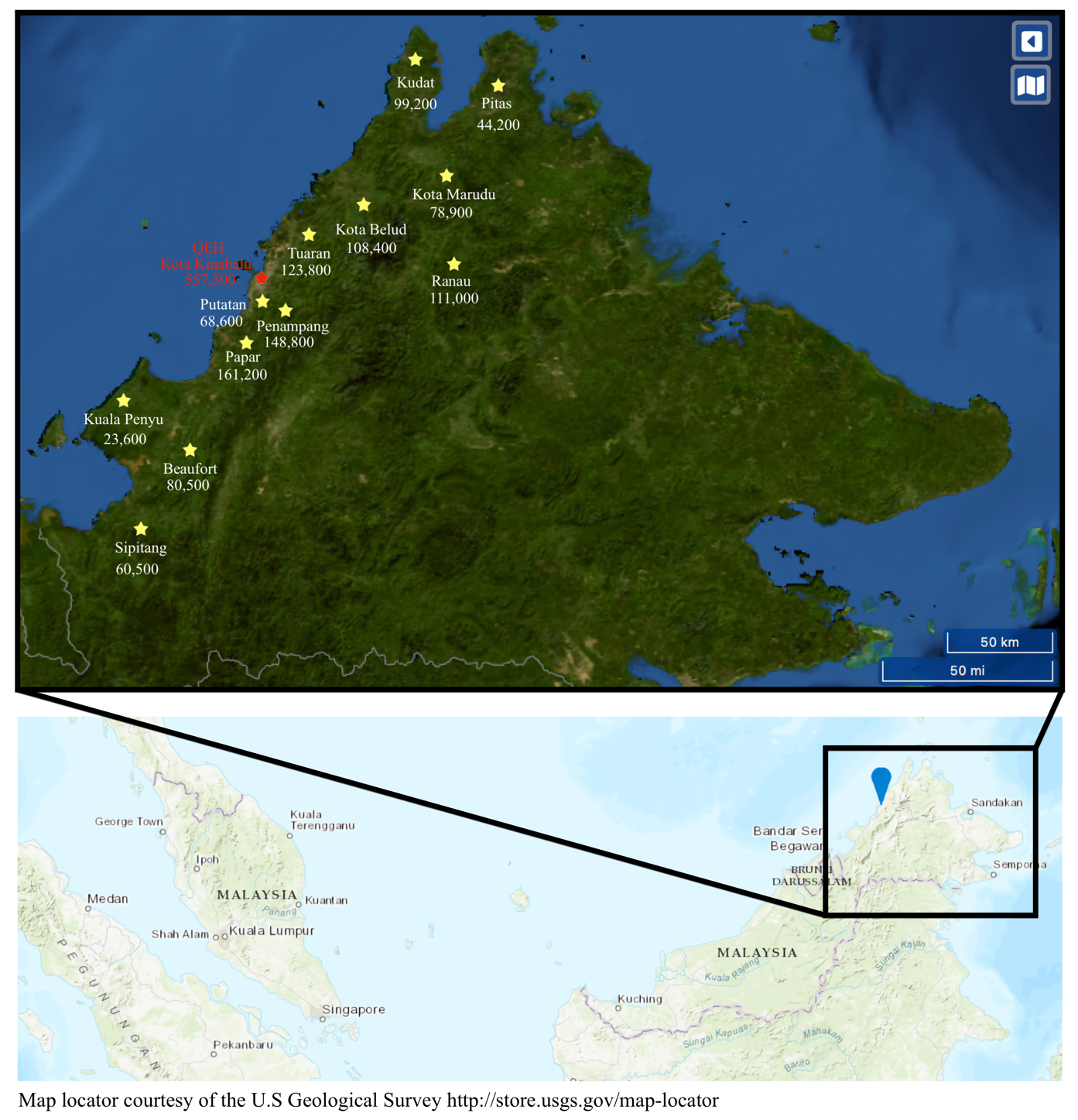


**S1 Fig QEH patients and specimens coverage by districts within west coast region of Sabah.** The map of Malaysia shows the Peninsular Malaysia and Malaysian states of Sarawak and Sabah in Malaysian Borneo region. The magnified view of Sabah map depicts the location of districts in Sabah (west coast region), covered by QEH with its population size(http://store.usgs.gov/map-locator).
