# Supplementary material for: Improving the clinical recognition, prognosis, and treatment of melioidosis through epidemiology and clinical findings: The Sabah perspective
Source: PLoS Negl Trop Dis. 2023 Oct 16;17(10):e0011696. doi: 10.1371/journal.pntd.0011696 (PMC10602235; doi:10.1371/journal.pntd.0011696)
Supplement: S1 Table — (DOCX) [file pntd.0011696.s002.docx]

**S1 Table Classification of the signs/symptoms based on the affected organ system**

| **Organ system affected** | **Signs/Symptoms** |
| --- | --- |
| Cardiovascular | Chest pain, heart attack, tachycardic |
| Musculoskeletal | Knee pain, joint pain, arthralgia, myalgia, shoulder pain, arthritis |
| Integumentary | Skin spot, rashes, skin abscess, skin carbuncle, gangrene, pus, blister, swelling, redness, necrotic patches, cellulitis, ulceration |
| Respiratory | Cough, bronchospasm, upper respiratory tract infection (URTI), pneumonia, shortness of breath (SOB), tachypnoea, respiratory distress, community-acquired pneumonia (CAP), hospital-acquired pneumonia (HAP). |
| Nervous | Meningitis, altered mental status, meningoencephalitis |
| Genitourinary | Acute kidney infection, haematuria, scrotum pain, prostatic abscess, renal abscess, urinary tract infection (UTI) |
| Lymphatic | Spleen abscess |
| Digestive | Peritonitis, transaminitis, abdominal pain, liver abscess, jaundice, epigastric pain |
