# Supplementary material for: Improving the clinical recognition, prognosis, and treatment of melioidosis through epidemiology and clinical findings: The Sabah perspective
Source: PLoS Negl Trop Dis. 2023 Oct 16;17(10):e0011696. doi: 10.1371/journal.pntd.0011696 (PMC10602235; doi:10.1371/journal.pntd.0011696)
Supplement: S2 Table — (DOCX) [file pntd.0011696.s003.docx]

**S2 Table Case fatality ratio and the odds ratio for death based on demographic data, symptoms and underlying illnesses among melioidosis culture-confirmed cases.**

|  | Melioidosis culture confirmed cases (*n* = 246) | | | | |
| --- | --- | --- | --- | --- | --- |
|  | *n* | Deaths (%) | OR | 95% CI |  |
| **Case fatality ratio**^a^ | 246 | 68 (28%) | - | - |  |
| **Gender** |  |  |  |  |  |
| Male | 188 | 56 (30%) | 1.63 | 0.80-3.33 |  |
| Female | 58 | 12 (21%) | - | - |  |
| **Age (years)** |  |  |  |  |  |
| <1 | 1 | 0 (0%) | - | - |  |
| 1-14 | 7 | 2 (29%) | 1.05 | 0.20-5.54 |  |
| 15-24 | 6 | 1 (17%) | 0.52 | 0.06-4.50 |  |
| 25-44 | 70 | 16 (23%) | 0.71 | 0.37-1.35 |  |
| 45-64 | 123 | 33 (27%) | 0.92 | 0.53-1.61 |  |
| 65+ | 37 | 15 (41%) | 2.00 | 0.97-4.15 |  |
| Unknown | 2 | 1 (50%) | 2.64 | 0.16-42.84 |  |
| **Ethnicity** |  |  |  |  |  |
| Malay | 20 | 6 (30%) | 1.13 | 0.42-3.08 |  |
| Chinese | 35 | 8 (23%) | 0.75 | 0.32-1.73 |  |
| Indian | 1 | 0 (0%) | - | - |  |
| Kadazan-Dusun | 83 | 25 (30%) | 1.20 | 0.67-2.16 |  |
| Other indigenous ethnic groups | 43 | 6 (14%) | 0.37 | 0.15-0.92 |  |
| Bajau | 37 | 13 (35%) | 1.52 | 0.72-3.19 |  |
| Murut | 7 | 2 (29%) | 1.05 | 0.20-5.54 |  |
| Non-Malaysians | 20 | 8 (40%) | 1.84 | 0.72-4.73 |  |
| **Bacteremia** | 227 | 67 (30%) | 7.54* | 1.00-57.61 |  |
| **Symptoms** ^b^ |  |  |  |  |  |
| Fever | 186 | 49 (26%) | 2.50 | 0.30-20.87 |  |
| Cardiovascular | 4 | 1 (25%) | 0.96 | 0.10-9.44 |  |
| Musculoskeletal | 22 | 5 (23%) | 0.83 | 0.30-2.38 |  |
| Integumentary | 27 | 6 (22%) | 0.80 | 0.30-2.11 |  |
| Respiratory | 160 | 47 (29%) | 4.30* | 1.25-14.75 |  |
| Nervous | 7 | 2 (29%) | 1.16 | 0.22-6.17 |  |
| Genitourinary | 19 | 7 (37%) | 1.79 | 0.66-4.84 |  |
| Lymphatic | 4 | 0 (0%) | - | - |  |
| Digestive | 47 | 7 (15%) | 0.42 | 0.18-1.02 |  |
| Unknown ^d^ | 52 | 18 (35%) | 1.53 | 0.79-2.94 |  |
| **Underlying illnesses** ^b^ |  |  |  |  |  |
| DM | 79 | 18 (23%) | 0.77 | 0.39-1.50 |  |
| Hypertension | 50 | 12 (24%) | 0.88 | 0.42-1.86 |  |
| CKD | 26 | 10 (38%) | 2.00 | 0.84-4.76 |  |
| Cardiovascular diseases | 15 | 6 (40%) | 2.05 | 0.69-6.07 |  |
| Thalassemia major | 2 | 0 (0%) | - | - |  |
| Malignancy | 3 | 1 (33%) | 1.45 | 0.13-16.33 |  |
| PTB | 7 | 4 (57%) | 4.09 | 0.88-18.94 |  |
| HIV | 1 | 1 (100%) | - | - |  |
| Hepatitis infection | 2 | 0 (0%) | - | - |  |
| Leptospirosis | 3 | 0 (0%) | - | - |  |
| **General health status** |  |  |  |  |  |
| Had at least one underlying illness | 106 | 26 (25%) | 0.87 | 0.46-1.65 |  |
| No underlying illness ^c^ | 88 | 24 (27%) | 1.15 | 0.61-2.20 |  |
| Unknown ^d^ | 52 | 18 (35%) | 1.52 | 0.79-2.94 |  |
| Total | 246 | 68 (28%) | - | - |  |
| **Patients’ outcome** |  |  |  |  |  |
| Septic shock | 55 | 26 (47%) | 4.30* | 2.16-8.55 |  |

OR, odds ratio; CI, confidence interval.

^a^ Data shown as case fatality ratio rather than the odds ratio for death.

^b^ Some patients may have had more than one symptom and/or underlying illnesses.

^c^ Including a patient who was less than 1-year-old.

^d^ Unable to acquire data during the study because of incomplete requisition forms.

*Indicates a *p*-value <0.05 (statistically significant).
